# *K-*Mer Analyses Reveal Different Evolutionary Histories of Alpha, Beta, and Gamma Papillomaviruses

**DOI:** 10.3390/ijms22179657

**Published:** 2021-09-06

**Authors:** Zigui Chen, Filippo Utro, Daniel Platt, Rob DeSalle, Laxmi Parida, Paul K. S. Chan, Robert D. Burk

**Affiliations:** 1 Department of Microbiology, Faculty of Medicine, The Chinese University of Hong Kong, Hong Kong, China; paulkschan@cuhk.edu.hk; 2 Centre for Emerging Infectious Diseases, Faculty of Medicine, The Chinese University of Hong Kong, Hong Kong, China; 3 Computational Genomics, IBM T. J. Watson Research, Yorktown Heights, NY 10598, USA; futro@us.ibm.com (F.U.); watplatt@us.ibm.com (D.P.); parida@us.ibm.com (L.P.); 4 Sackler Institute of Comparative Genomics, American Museum of Natural History, New York, NY 10024, USA; desalle@amnh.org; 5 Department of Pediatrics, Albert Einstein College of Medicine, Bronx, NY 10461, USA; 6 Department of Microbiology and Immunology, Albert Einstein College of Medicine, Bronx, NY 10461, USA; 7 Department of Epidemiology and Population Health, Albert Einstein College of Medicine, Bronx, NY 10461, USA; 8 Department of Obstetrics, Gynecology and Woman’s Health, Albert Einstein College of Medicine, Bronx, NY 10461, USA

**Keywords:** *k*-mer distribution, *Papillomaviridae*, evolution, Alpha-papillomavirus, Beta-papillomavirus, Gamma-papillomavirus

## Abstract

Papillomaviruses (PVs) are a heterogeneous group of DNA viruses that can infect fish, birds, reptiles, and mammals. PVs infecting humans (HPVs) phylogenetically cluster into five genera (Alpha-, Beta-, Gamma-, Mu- and Nu-PV), with differences in tissue tropism and carcinogenicity. The evolutionary features associated with the divergence of *Papillomaviridae* are not well understood. Using a combination of *k*-mer distributions, genetic metrics, and phylogenetic algorithms, we sought to evaluate the characteristics and differences of Alpha-, Beta- and Gamma-PVs constituting the majority of HPV genomes. A total of 640 PVs including 442 HPV types, 27 non-human primate PV types, and 171 non-primate animal PV types were evaluated. Our analyses revealed the highest genetic diversity amongst Gamma-PVs compared to the Alpha and Beta PVs, suggesting reduced selective pressures on Gamma-PVs. Using a sequence alignment-free trimer (*k* = 3) phylogeny algorithm, we reconstructed a phylogeny that grouped most HPV types into a monophyletic clade that was further split into three branches similar to alignment-based classifications. Interestingly, a subset of low-risk Alpha HPVs (the species Alpha-2, 3, 4, and 14) split from other HPVs and were clustered with non-human primate PVs. Surprisingly, the trimer-constructed phylogeny grouped the Gamma-6 species types originally isolated from the cervicovaginal region with the main Alpha-HPV clade. These data indicate that characterization of papillomavirus heterogeneity via orthogonal approaches reveals novel insights into the biological understanding of HPV genomes.

## 1. Introduction 

Papillomaviruses (PVs) are a heterogeneous set of double-stranded DNA viruses with a circular genome of approximately 8000 bp. PVs mainly infect epithelial surfaces, for example, anogenital skin and mucosa, of a large spectrum of host species including humans, monkeys, reptiles, birds, aquatic, and terrestrial vertebrates, and have co-evolved with their host species for millions of years [1,2]. PV infections are primarily asymptomatic, although some cause benign flat or protruding warts. In contrast, persistent infection of high-risk human papillomaviruses (such as HPV types 16 and 18) is associated with cervical cancer as well as cancers of the lower genital tract and head and neck regions [3]. PV genomes typically contain eight open reading frames (ORFs) that encode core and accessory viral proteins [4]. Core proteins include two early products (E1 and E2) and two late products (L1 and L2), are highly conserved among all papillomaviruses and are involved in viral genome replication and viral assembly, respectively. By contrast, the accessory proteins (E5, E6 and E7) show greater variability in gene expression and function and are not present in all PV types. Some E5, E6, and E7 proteins are also associated with cell immortalization and transformation. After establishing infection by accessing the epithelial basal layer, PV genes are regulated by multiple promoters and a complex patterns of mRNA splicing that coordinates viral gene expression with different stages of the viral life cycle [5].

The PV genome has served as the basis for classification and understanding the biology and disease association of this ancient and diverse set of viruses. The designation of a PV type represents the fundamental taxonomic level of a highly related group of viruses that have similar genetic and biologic characteristics [6,7,8]. Currently, over 200 human papillomavirus (HPV) types and 160 non-human animal PV types have been officially characterized and curated (http://www.ictvonline.org/; accessed on 31 October 2018) [9]. HPVs cluster into five phylogenetic genera (designated Alpha-, Beta-, Gamma-, Mu-, and Nu-PV) out of at least 53 known genera [8]. HPV genera, species and/or types have been noted to be adapted to specific human epithelial niches, with differences in cell tropism and disease associations. For example, all genital high-risk HPV types causing cervical cancer are part of a monophyletic clade within the genus Alpha-PV. Beta-HPVs have been associated with squamous cell cancer of the skin, particularly in subjects with epidermodysplasia verruciformis (EV); Beta- and Gamma-HPVs are also commonly detected in healthy skin swabs and oral cavity samples [10,11,12]. The relative tissue tropism of the HPV genera was considered to be a manifestation of niche adaptation [13]. However, with the development of sensitive assays employing PCR amplification and more recently, metagenomic analyses, it has become clear that a number of novel and characterized HPV types are widely detected in different anatomic regions including genital, oral and skin epithelium [14,15,16,17,18,19,20,21], indicating that the tissue-tropism of HPVs is not completely understood. For example, several studies have demonstrated the association of HPV16 (representing infections in >50% cervical cancers) with head and neck squamous cell carcinomas (HNSCC) specifically oral and oropharyngeal squamous cell carcinomas [22,23,24,25]. The genus Alpha-PV also contains low-risk human viruses causing anogenital and skin warts (for example, HPV6 and HPV11) [26] as well as macaque viruses isolated from the monkey cervicovaginal region that are also associated with cervical cancers (for example, MfPV3) [27,28]. Thus, the imperfect boundary between clinical manifestation and phylogenetic relationships for HPVs may imply complex evolutionary histories and interactions between viruses and their hosts undergoing natural selection, niche adaptation, host immune response, and/or viral recombination [29]. 

Characterizing potential mechanisms driving genetic diversity upon which selection and fixation occurs is important for understanding viral associated diseases and basic principles of genome evolution. Since PVs rely on host enzymes for viral gene expression and replication, virus-host biological interactions are critical for viral fitness. It has been reported, for example, that HPVs have a strong codon usage bias different from their human hosts [30,31]. Codon usage bias in the viral genome is often explained by evolutionary adaptation to the host immune defenses through processes such as immune evasion (by decreasing viral protein synthesis) and a tightly regulated viral life cycle (by optimizing viral infection in the differentiating keratinocyte) [32]. Human innate immune system proteins, such as APOBEC, may act as restriction factors for HPV infection and can lead to a mutational signature in the viral genome associated with increased fitness [33,34]. 

With advances in DNA sequencing and bioinformatics analyses, multiple sequence alignments have been used to interrogate PV genomes using parametric methods. For example, incongruent tree topologies observed between early and late gene phylogenies suggested potential recombination events in Alpha-, Omicron- and Upsilon-PVs implying that rare events may contribute to the complex evolutionary history in part obscuring simple host–parasite coevolution [35,36,37,38]. The highly divergent homologous sequences among PV genomes, however, can lead to ambiguous alignments that obscure resolution and bias phylogenetic inferences. Alternatively, nonparametric agnostic approaches based on the distribution of exact sub-sequences (*k*-mer: a DNA ‘word’ with fixed length) and additional genetic metrics (for example, codon usage preference, dimer nucleotide composition) provide complementary information on the complexity and relationships of PV genomes [38,39,40,41,42]. These agnostic methods also avoid complex computation or model selection while capturing signals otherwise lost to indel, recombination or genome shuffling [41,43]. 

In an effort to interrogate evolutionary features associated with the divergence of papillomaviruses generally, and HPVs specifically, we analyzed genomic features between Alpha-, Beta-, and Gamma-PVs constituting the majority of HPV genomes. We have primarily focused on PVs from a single host (human) to avoid strong host biases, and to try to observe genomic features possibly associated with phenotypic outcomes such as cancer. Multiple parametric and nonparametric algorithms were applied. Using the characterization of a large number of PV genomes, we sought to uncover hidden biological signals not easily discovered with homology-based methods alone.

## 2. Results 

### 2.1. Phylogeny of Human Papillomaviruses Based on Homology 

**Figure 1:**
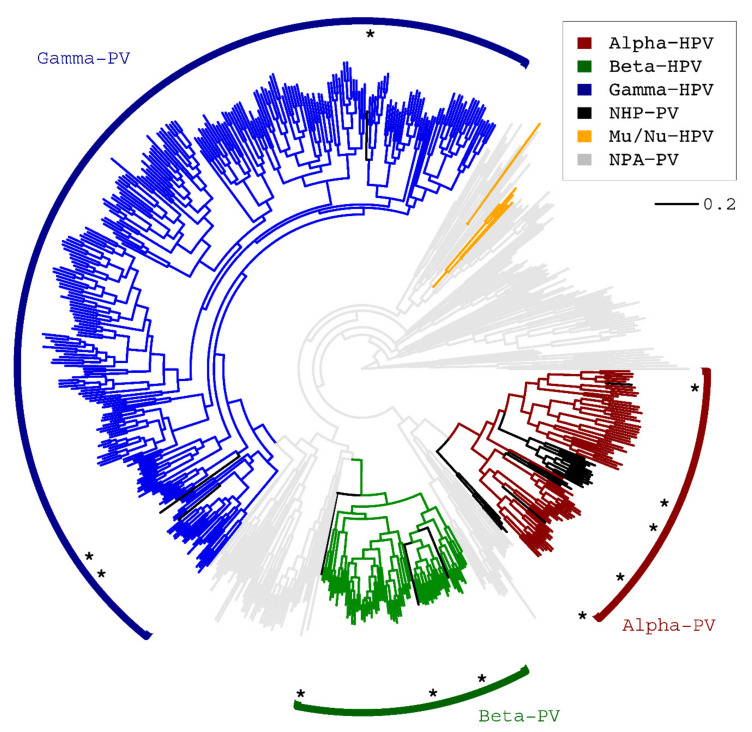
Homology-based phylogeny of papillomaviruses. A maximum likelihood (ML) tree was constructed using RAxML MPI v7.2.8 inferred from the concatenated nucleotide sequence alignments of E1-E2-L2-L1 open reading frames (ORFs) of 640 PV types. All characterized HPVs are contained within the genera of Alpha-, Beta-, Gamma-, Mu-, and Nu-PV. The black lines and stars indicate non-human primate PV (NHP-PV) species clustering within the genus Alpha-, Beta-, Gamma- and Dyoomikron-PV. Non-primate animal PVs (NPA-PVs) are indicated by grey lines. The bar indicates the nucleotide substitution of unit changes (i.e., 0.2) per site.

To better understand the evolutionary history and genetic diversity of human papillomaviruses, we first assessed the phylogeny of the main subfamily, *Firstpapillomavirinae* within the family *Papillomaviridae* [7] that included 442 human PV (HPV) types, 27 non-human primate PV (NHP-PV) types and 171 non-primate animal PV (NPA-PV) types (a total of 640 PV types, dataset 1) (Table S1). The phylogenetic tree inferred from the core homologous codon sequences clustered the majority of HPVs (98.6%, 436/442) into three distinct genera including Alpha-, Beta- and Gamma-PV, as expected (Figure 1). Beta- and Gamma-PVs shared a most recent common ancestor and are relatively distant to Alpha-PVs, which is consistent with the tissue tropism of the anatomic sites from which the viral types were originally isolated. NHP-PVs shared a similar phylogenetic topology with their human counterparts, as all characterized Old World monkey and ape PVs (e.g., macaque PVs, Colobus monkey PVs, chimpanzee PV) clustered into Alpha-, Beta- and Gamma-PVs (indicated types with black lines and species marked with asterisks in Figure 1). At least 14 (α1-14), 6 (β1-6) and 27 (γ1-27) species groups have been identified within the genera Alpha-, Beta- and Gamma-PV, respectively, while the genus Gamma-PV contains a higher genomic heterogeneity (inter-species difference of 41.9 ± 1.8%) compared to Alpha- (39.7 ± 2.1%, *p* < 0.001) and Beta-PVs (35.9 ± 1.4%, *p* < 0.001) (Figure S1A). Distinct ORFs have a high level of genomic heterogeneity (Figure S1B). The major capsid protein (L1) (*pi* = 0.37 ± 0.03) and the multipurpose DNA helicase E1 (*pi* = 0.40 ± 0.04) were the most conserved genes compared to other genes (0.53 ± 0.06 > *pi* > 0.43 ± 0.04). Alpha-HPVs are constituted by three monophyletic branches including one high-risk clade (Alpha-HPV-HR: α5, 6, 7, 9 and 11) and two low-risk clades (Alpha-HPV-LR1: α1, 8, 10 and 13; Alpha-HPV-LR2: α2, 3, 4 and 14) (Figure S2, Table S2). 

### 2.2. Trimer Clustering of Human Papillomaviruses 

**Figure 2:**
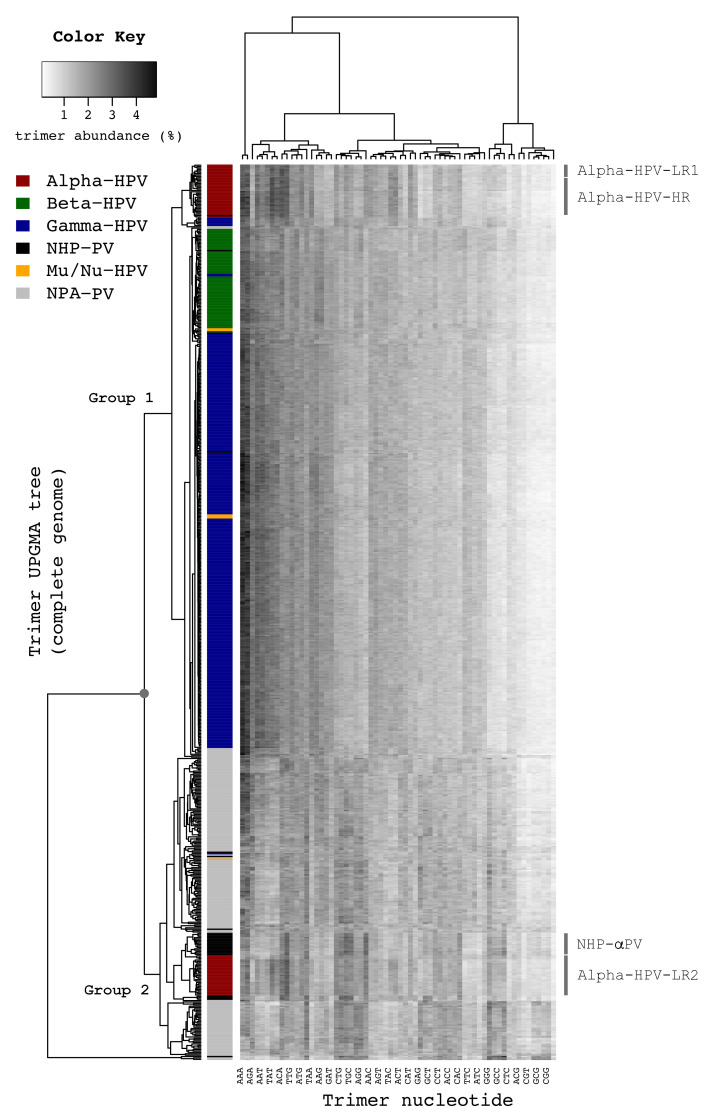
Phylogeny of papillomaviruses based on trimer distributions in papillomavirus sequences. The proportional abundances (%) of each trimer (61 trimers except for TAG, TAA, TGA, shown at the bottom of the heatmap) in 640 PV types were constructed based on the complete genome nucleotide sequences as a heat map scaled in black to white gradients, with white indicating minimal probability (0.14%) and black indicating the maximum probability (4.83%). The dendrogram to the left of the figure was generated using hierarchical clustering based on the trimer distribution Kullback-Leibler (KL) distances. The grey circle on the tree indicates the node split between groups 1 and 2.

In order to explore an agnostic evolutionary model of HPV genomes, we constructed a phylogeny using trimer (*k* = 3) spectra. This analysis has the potential to reveal “higher order” features of viral genomes in comparison with the alignment-based phylogeny that relies primarily on protein coding regions [44]. As shown in Figure 2, nearly all PV genomes (except for AgPV1 within the genus Dyoomikron-PV and, AmPV3 and ZcPV1 within Dyonu-PV) divided into two main groups, with deep separation between viruses mainly infecting human compared to non-human hosts (see Table S2 for genome types and their hosts). The majority of HPV genomes (93.0%, 411/442), including all 26 Alpha-HPV-HR types, 11 of 12 Alpha-HPV-LR1 types, 69 Beta-HPV types, 300 of 301 Gamma-HPV types, and 5 of 6 Mu/Nu-HPV types, grouped together and formed three distinct clades within Group 1, implying that HPVs sharing a single host (human) generally have similar trimer distributions. Group 1 also contained 4 non-human primate PV types (PpPV1 within the species α10, MfPV1 within the species β1, MmPV5 and 7 within the genus Gamma-PV) and 9 non-primate animal PV types from a wide range of host species (see Table S2). 

**Figure 3:**
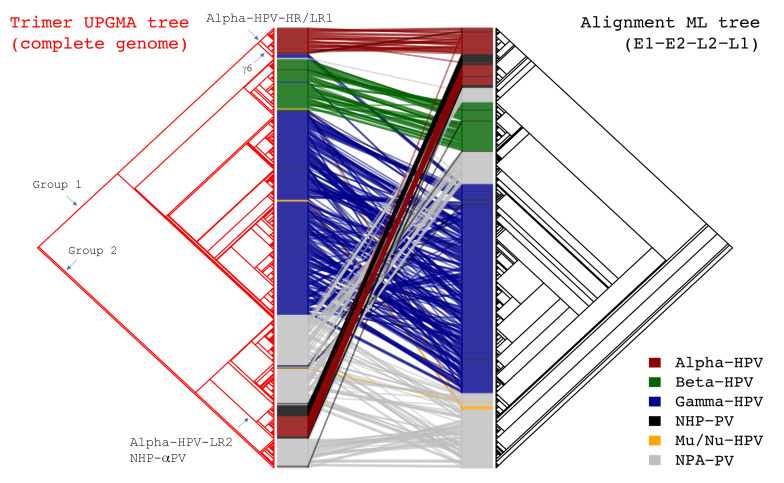
Tanglegram of tree topologies between phylogenies based on the trimer distribution KL distances and the maximum likelihood (ML) tree inferred from the concatenated nucleotide sequence alignments of 4 ORFs (E1-E2-L2-L1). 640 PV genomes are included. The bar to the side of each panel indicates the genus assignment of each PV type and is colored according to the key in the figure.

Remarkably, HPV types from the Alpha-HPV-LR2 clade (α2, 3, 4 and 14) separated from the main cluster of HPVs and sorted to the clade containing the majority of non-human primate PVs (in black) and non-primate animal PVs (in grey) within Group 2 (Figure 2 and Figure 3). Although evolutionary incongruence of PV genera inferred from different genes has been reported [2,36], we found that the majority of genomes maintained a consistent clustering using either early (E1-E2) or later genes (L2-L1) when based on the trimer distribution (Figure S3A). Interestingly, the species γ6 (HPV101, 103, 108, 214, 226 and w02c24a) clustered at the root of the Alpha-HPV-HR/LR1 clade based on the trimer distribution (Figure 3). The species γ6 has been reported to be commonly found in cervicovaginal samples and lesions, yet lacks a canonical E6 ORF [45,46]. Thus, the trimer spectra phylogeny placed these Gamma-HPV types in a set of papillomaviruses with similar tissue tropism. Overall, the matrix used for the trimer phylogeny showed a strong correlation with the matrix used for the homology-based phylogeny; a value of zero would indicate no correlation (Mantel test = 0.4551, *p* < 0.001) (Figure S3B).

### 2.3. Codon Usage Biases of HPV Genomes

**Figure 4:**
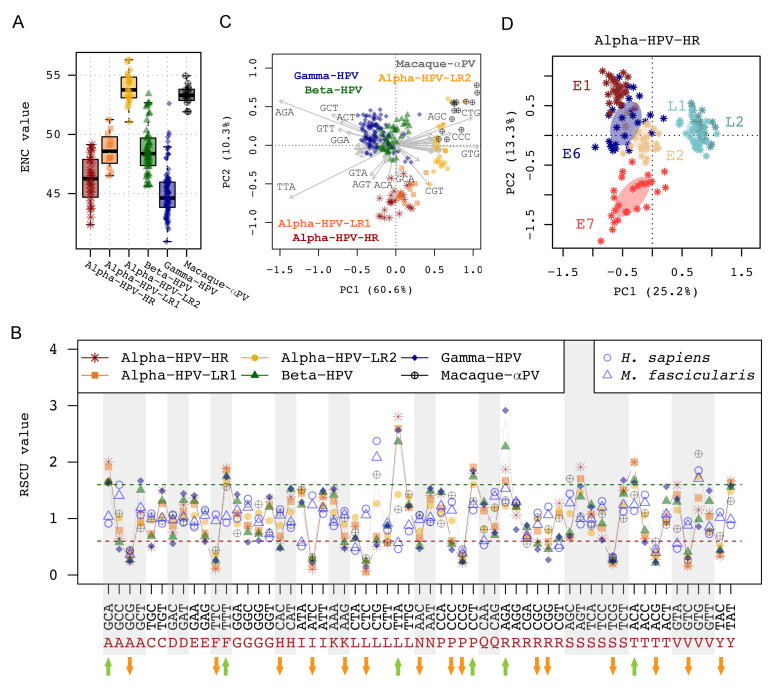
Synonymous codon usage of 228 PV genomes based on the concatenated nucleotide sequences of 6 ORFs (E6-E7-E1-E2-L2-L1). (**A**) Boxplot of Effective Number of Codon (ENC) between PV groups/genera. The ENC values range from 20 when a gene is effectively using only a single codon for each amino acid (strongest bias) to 61 when a gene uses all codons with equal frequency (no bias). (**B**) Mean values of Relative Synonymous Codon Usage (RSCU) for 59 codons (except for Met, Trp, and stop codons) amongst PV groups/genera. The preferred and suppressed codon usages were defined if RSCU values were >1.6 or <0.6, respectively. Those codons with increased or decreased RSCU values are shown indicated with green or orange arrows at the bottom of the figure, respectively. (**C**) Scatter plot of RSCU difference of PV genomes. The clustering was performed using redundancy analysis (RDA), with colors assigned to different PV groups/genera. The x-axis and the y-axis represent the first two principal coordinate component (PC) axes. (**D**) Scatter plot of RSCU difference of PV genes within the HR clade of the genus *Alphapapillomavirus*. The clustering was performed using redundancy analysis (RDA), with colors assigned to different PV genes. The x-axis and the y-axis represent the first two principal coordinate component (PC) axes.

Since many trimers are also codons, the differences in the trimer distributions between genomes might imply differential codon usages between viruses and host epithelial cells. To estimate this possibility, we measured the effective number of codons (ENC) values across 6 open reading frames (ORFs) to compare the codon usage bias between different HPV phylotypes (Alpha-HPV-HR, Alpha-HPV-LR1, Alpha-HPV-LR2, Beta-HPV, and Gamma-HPV) [47]. Only officially assigned HPV types were included, given their highly curated ORF sequences (N = 214, dataset 2, Tables S1 and S2). In addition, fourteen macaque Alpha-PVs were also included for comparison. The ENC values of the surveyed HPV genomes ranged between 41.0 and 56.3, with a mean value of 47.4, indicating that the overall codon usage bias was not very extreme (Figure 4A). In general, a gene with an ENC less than 36 is thought to possess strong codon bias [48]. We found significantly higher ENC values amongst the Alpha-HPV-LR2 clade when compared to other HPVs (a mean of ENC values 54.0 ± 1.3 vs. 46.5 ± 2.6, *p* < 0.001). In contrast, Gamma-HPVs had the lowest ENC values (45.1 ± 2.0), followed by Alpha-HPV-HR (46.2 ± 2.0), Alpha-HPV-LR1 (48.7 ± 1.5) and Beta-HPV (48.7 ± 2.0), although a phylogenetic generalized linear model test (*pglm*) did not support statistical significance between HPV groups (*p* = 0.1621). Consistent with the trimer distribution clustering, macaque Alpha-PVs codon usage did not differ from the Alpha-HPV-LR2 types (53.3 ± 0.8 vs. 54.0 ± 1.3, *p* = 0.0938) (see Figure 2).

To pursue explanations for the trimer distribution analysis tree, we looked at the relationship between codon usage bias and ORF sequence composition. As shown in Figure S4A, a plot of ENC values against GC3s (GC content at synonymous third codon position) found that all surveyed PV genomes lay slightly under the expected ENC* curve (the red curve), suggesting that the codon usage pattern may be affected by GC3s and is likely subject to GC-biased mutational pressure [49]. It is worth noting that other evolutionary factors such as translational or natural selection may also act as additional forces influencing the differential codon usages observed between PV genomes infecting macaques and human beings, since a higher measure of (ENC* ENC)/ENC* in macaque PVs indicated the codon usage bias tended to be less dependent on variation of GC3s when GC contents increased (Figure S4B). However, codon usage bias seems highly variable between genes/ORFs, as measured by both ENV values and the results of (ENC* ENC)/ENC* analyses (Figure S4C,D). 

### 2.4. Synonymous Codon Usage Pattern in Human Papillomaviruses

Besides the codon usage measurement of an entire genome and/or gene, we calculated the Relative Synonymous Codon Usage (RSCU) values of HPV genomes. This analysis estimates the differential usage of each synonymous codon. Among 59 codons encoding 18 amino acids (excluding codons for Met, Trp, and stop codons), 6 codons were preferred (RSCU values > 1.6, green arrow pointing up) and 15 were suppressed (<0.6, orange arrow pointing down) across the surveyed PV genomes (Figure 4B, Table 1). Most HPV-preferred codons are not commonly found in human genes. For example, the RSCU value of TTA encoding Leucine (Leu) was higher (2.40) in HPV genomes, compared to ORFs in human genomes (0.46). In contrast, CTG, the preferred codon for Leu in the human genome (2.37), was rarely found in viral genomes (0.65). Interestingly, 26 out of 29 HPV codons (90%) with RSCU > 1.0 ended with A/T, indicating that HPV genomes intensely use A/T-ending codons while their hosts (human and macaque) use more G/C-ending codons (68%, 17/25). HPV genomes also displayed a strong tendency to avoid using nCG or nTC codons (RSCUs < 0.31); whereas, in human genes NCGs (mean RSCU value of 0.41) were also suppressed, but not NTCs (RSCU value 1.15).This suggests a potential role of CpG dinucleotide depletion in shaping the codon usages of both HPVs and their host.

### 2.5. Analysis of Synonymous Codon Usage 

To investigate the role of synonymous codon usage bias in modulating HPV genomic diversity, a correspondence analysis based on RSCU patterns was performed [50]. Scatter plots of the first two axes supported the clustering of the surveyed PV genomes into three groups mainly composed of Alpha-HPV-HR/LR1, Beta-/Gamma-HPV, and Alpha-HPV-LR2/Macaque-αPV (Figure 4C). This observation was evaluated with a permutational multivariate analysis of variance (PERMANOVA), which indicated that approximately 58% of variation in synonymous codon usage bias (Df = 2, R^2^ = 0.5773, pseudo F = 150.2, *p* < 0.001) was attributable to the differences between PV groups. Beta-/Gamma-HPV genomes predominantly infecting the skin regions clustered together (i.e., shared similar codon usage patterns to each other), with a tendency to use more AGA encoding for Arginine when compared to the mucosal types (Figure 4B,C, Table 1). Alpha-HPV-LR2/Macaque-αPV groups shared relatively similar codon usage patterns mimicking the host species, using AGC, CCC, CTG and GTG encoding for Serine, Proline, Leucine and Valine, respectively. The extent of relative synonymous codon usage also varied between HPV genes (Figure S5, Tables S3 and S4). For example, the E1 (R^2^ = 0.4295) and L1 (R^2^ = 0.3894) genes contained more variation in synonymous codon usage that results in the clustering of HPV taxa, followed by L2 (R^2^ = 0.3761), E2 (R^2^ = 0.2253), E6 (R^2^ = 0.2082) and E7 (R^2^ = 0.1120) (Figure S5). HPV genes with similar biological functions such as temporal expression may share codon usage patterns (Figure 4D and Figure S6). In addition, L1 and L2 genes encoding PV capsid proteins firmly clustered together in codon usage pattern. In contrast, the oncogenic protein E7 was separated from the other genes. 

### 2.6. Dinucleotide Suppression in Human Papillomavirus Genomes 

**Figure 5:**
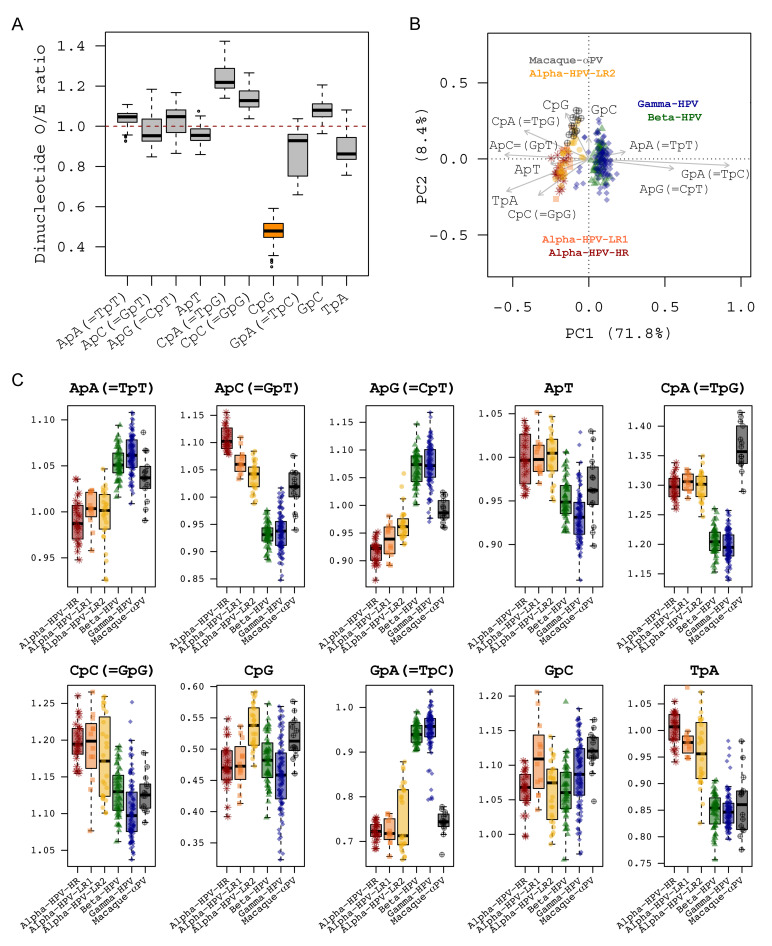
Dinucleotide suppression within 228 PV genomes based on the concatenated nucleotide sequences of 6 ORFs (E6-E7-E1-E2-L2-L1). (**A**) Boxplot of dinucleotide observed/expected (O/E) ratio. The XpY dinucleotide exhibits suppression if the O/E ratio is less than 1. (**B**) Scatter plot of relative abundance of dinucleotides among PV genomes. The clustering was performed using redundancy analysis (RDA), with colors assigned to different PV groups/genera. The x-axis and the y-axis represent the first two principal coordinate component (PC) axes. (**C**) Boxplot of the O/E ratios of each dinucleotide amongst PV groups/genera.

To investigate the influence of dinucleotide suppression on codon usage bias and genomic variability we measured the relative abundance of different dinucleotides across the genomes. As expected, the CpG dinucleotide was mostly depleted when compared to other dinucleotides (mean value of observed/expected ratio of 0.48 ± 0.05) (Figure 5A, Table 2 and Table S5), consistent with the low abundance of NCG codon usages (Figure 4B, Table 1 and Table S3). The GpA (=TpC in the complementary strand) represented the second most suppressed dinucleotide (0.87 ± 0.11), consistent with the scarcity of NTC codon usages as well. Since GpA (=TpC) dinucleotide is one of the preferred target sequences of host restriction factor APOBEC3 proteins, its suppression could be a result of evolutionary selection, allowing viruses to evade restriction from host innate immune mechanisms [51]. The TpA dinucleotide was also underrepresented in PV genomes (0.89 ± 0.07), probably due in part to usage of universal stop codons (TAA, TAG) and the increased susceptibility of TpA to ribonuclease digestion [52]. We found discriminative patterns of dinucleotide suppression between Alpha-HPVs and Beta-/Gamma-HPVs (Figure 5B), probably implying an evolutionary apomorphy in association with viral tissue tropism. Notably, GpA (=TpC) dinucleotides were significantly depleted in Alpha-HPV genomes compared to Beta-/Gamma-HPVs (0.74 ± 0.05 vs. 0.95 ± 0.04, *p* < 0.001) (Figure 5C, Table 2). In contrast, Beta-/Gamma-HPVs had lower O/E ratios of ApC (=GpT) (0.94 ± 0.03 vs. 1.07 ± 0.04, *p* = 0.004) and TpA (0.85 ± 0.03 vs. 0.98 ± 0.05, *p* = 0.007). We also observed substantial dinucleotide suppression between HPV genes suggesting that phenotypes and proteins sharing similar biological properties were often clustered (Figures S7 and S8, Table 2, Tables S5 and S6). 

### 2.7. Association between Dinucleotide Suppression and CpG Methylation

**Figure 6:**
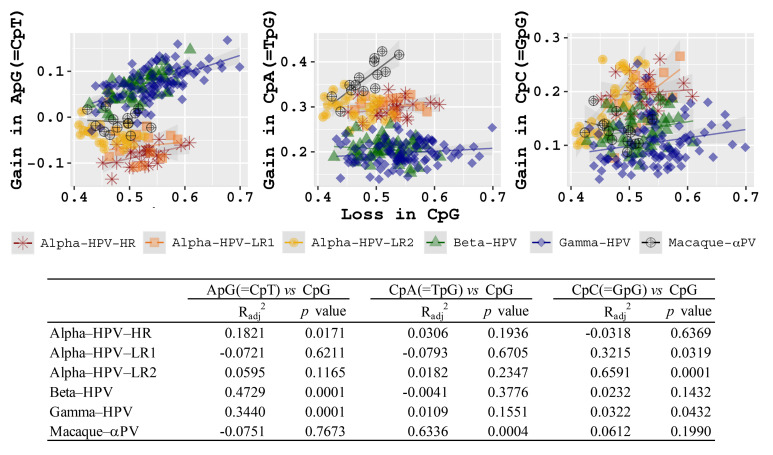
Correlations between the loss in CpG and the gain in non-CpG potential methylation dinucleotides (ApG = CpT, CpA = TpG, CpC = GpG) inferred from the concatenated nucleotide sequences of 6 ORFs (E6-E7-E1-E2-L2-L1) of 228 PV types. The correlation test was performed within each PV groups/genera, with adjusted R^2^ and *p* values listed below the scatterplots.

It has been reported that depletion of CpG dinucleotide could be the result of deamination of 5-methylcytosine (5mC) leading to a cytosine (C) to thymine (T) transition within a CpG island, resulting in a gain of CpA (=TpG) [53]. Methylated cytosines are found primarily at CpG dinucleotide, but can also be found at non-CpG sites, including CpA (TpG), CpT (ApG), and CpC (GpG) that are frequent in HPV genomes. Interestingly, significant associations between the loss of CpG dinucleotide and the average gain of potential non-CpG methylation dinucleotides (ApG = CpT, CpA = TpG, CpC = GpG) were observed within certain HPV groups and/or genes (Figure 6). For example, within Beta-/Gamma-HPVs there was a strong correlation between the loss of CpG and the gain of ApG (=CpT) (*p* < 0.001). The loss of CpG in Macaque-αPV and Alpha-HPV-LR2 genomes appeared to be positively associated with the gain of CpA (=TpG) and CpC (=GpG), respectively. The data suggest a potential role of the deamination of methylated C to T in CpG dinucleotide depletion, but mutational pressure, genetic drift and/or translational selection, could also be driving CpG suppression in HPV genomes.

## 3. Discussion

In this study, we applied multiple parametric and nonparametric algorithms including evolutionary phylogeny, trimer distributions, codon usage bias and dinucleotide suppression to systematically evaluate HPV genomic characteristics. Using the sequences of a large number of viral genomes, we provide evidence of differential evolutionary constraints associated with the heterogeneity of HPV genomes. Using an agnostic trimer analysis, we show that the Alpha-PVs are more disparate than assumed from homology-based analyses. In particular, the separation of a low-risk Alpha-HPV clade (i.e., Alpha-HPV-LR2) from the main clustering of HPV genomes was consistently supported by trimer phylogeny, codon usage and dinucleotide suppression. These observations suggest that additional information in papillomavirus genomes, revealed by these analyses, could be under evolutionary pressures and contribute to HPV niche adaptation, host specificity, immune evasion, and pathogenicity [54,55,56]. 

HPVs were assumed to have evolved from multiple ancestors adapted to the epithelial cells of different host ecosystems prior to the speciation events of primate host species [13]. Following periods of adaptation to more specific host niches, phylogenetically related viruses were transmitted within similar ecosystems, resulting in the radiation observed in the phylogenetic tree in which viruses with similar tropism, biological properties and clinical potential usually cluster together. However, the genetic heterogeneity, such as recombination, duplication, insertion/deletion, genetic fusion and shuffling, and potential sequencing errors, challenges the accuracy of sequence alignments and comparisons that strongly relies on heuristic solutions and data quality. The alignment of PV genomes with complex evolutionary histories introduces uncertainty in analyses based on homology [2,57]; hence, complementary approaches provide an opportunity to further explore the evolution of *Papillomaviradae*. Our data indicated that *k*-mer distribution (*k* = 3), codon usage bias and dinucleotide suppression allow statistical analyses to identify sequence signatures of PV genomes corresponding to differences in host specificity (human vs macaque PVs), tissue tropism (mucosal vs cutaneous HPVs), and carcinogenic potential (high-risk vs low-risk HPVs). Viruses infecting a single host (e.g., human) have a tendency to contain similar genetic features. Within the genus *Alphapapillomavirus*, for example, we found two deeply separated groups (i.e.,Alpha-HPV-HR/LR1 and Alpha-HPV-LR2/Macaque-αPV) with significant differences in trimer distributions, codon usage and GC content. Strikingly, Gamma-6 (γ6) HPV types shared more “high order genomic features” with Alpha mucosal HPVs (see Figure 3). This is an example of genome content analysis using trimer distributions that places a clade into a set of viruses with a similar tissue tropism that was not appreciated from homology-based analyses.

We found different patterns of codon usage bias and dinucleotide suppression between HPV late and early genes, and between core and accessory genes, in line with previous observations [31,58]. The E7 gene was distinguished from other genes based on codon usage patterns. The difference in codon usage bias between HPV genes might be explained in part by mutational pressure and selection on genes with different expression levels and/or lengths, since selection may be acting to maximize translational efficiency of energetically costly longer genes and reduce the size of highly expressed genes [30,59].

It has been reported that codon usage preferences among organisms reflect a balance between mutational biases, genetic drift and natural selection for translational optimization [60,61]. We observed significantly different codon usage patterns in HPV genomes compared to that of the human host genome, with a correlation between GC content and codon usage bias. The results suggest that variation in mutational bias is a major force shaping codon usage, as proposed in previous studies and found with other vertebrate DNA viruses [31,42,62]. However, other independent factors, such as translational or natural selection, could act as modifiers underlying synonymous codon usage in the HPV genome or genes. 

Papillomaviruses rely on host cellular machinery for transcription and replication; codon usage biases in HPV genomes could be explained as an evolutionary adaptation to host defenses. Firstly, deoptimized codon usage in HPV genomes with respect to that of its cellular host may facilitate viral fitness by limiting viral gene expression and eliciting host immune responses [63,64]. Wild-type HPV genes expressed in human cell culture usually produce low amounts of protein; whereas, high levels of viral gene expression have been achieved when the gene sequences were optimized to those more common in human genes [65,66,67]. It was assumed that decreased protein synthesis of HPV genes lead to a less intense host immune response. For example, expression of L1 and L2 genes, the most immunogenic proteins, are only detected in the most terminally differentiated keratinocytes [68]. Suppression by means of codon usage maladaptation may allow viruses to better escape immune surveillance for persistence of infection. In contrast, the E7 gene with codon usage more compatible with the average human codon usage preferences usually yielded enhanced expression of protein [69,70]. Secondly, codon usage in HPV genes might be subject to host innate immune pressure, such as from ABOPEC3, a family of cellular cytidine deaminases that introduce directional C>T substitutions [71]. It has been reported that APOBEC3-mediated cytidine deaminase activity could target HPV genes to eliminate and/or mutate transfected viral DNA. These induced mutations, if not lethal, may also be responsible for the long-term accumulation of genomic changes that affect the success of niche adaptation or functions that contribute to HPV-associated cancer [72]. Interestingly, TpC (=GpA) dinucleotide and TpC-ending codons, the preferred dinucleotide target site of many APOBEC3 members, were dramatically underrepresented in HPV genomes. However, it has been found that the TpC suppression was most dramatic amongst the mucosal Alpha-HPVs and not cutaneous Beta-/Gamma-HPV types [51], consistent with the observation of significantly highly expressed APOBEC3 isoforms in mucosal skin compared to cutaneous skin [73], resulting in differential depletion of TpC dinucleotides relevant to tissue tropism. Thirdly, codon usage and dinucleotide composition of HPV genomes could be partially due to host innate antiviral activities including DNA methylation. Methylation on cytosine, facilitated by host cellular methyltransferases, has a tendency to undergo deamination when unpaired, resulting in the mutation of C to T [42,74]. DNA methylation functions as a host defense mechanism by regulating gene expression. When CpG residues of foreign DNA are methylated, pathogen activity can be repressed due to alterations in pathogen transcriptional profiles. CpG motifs in HPV genomes can be methylated. For example, HPV gene transcription is repressed by hypermethylation of the viral upstream regulatory region (URR) [75]. Numerous studies, primarily on the high-risk Alpha-HPV L1 and L2 regions, have documented that CpG methylation levels change not only in the context of the viral life cycle, but also in the context of HPV-lesion progression to cancer [76,77,78,79]. Similar to a number of other small viruses, the frequency of CpG dinucleotide is markedly reduced in HPV genomes [51,53,74,80], which could be tied to evolutionary selection in regulating gene expression, assisting immune evasion, and avoiding C to T mutation elicited by host DNA methylation. However, the extent and dynamics of CpG methylation in HPV genomes and the role in vegetative viral replication and progression to disease warrants further study.

## 4. Materials and Methods

### 4.1. Papillomavirus Complete Genome Dataset

We retrieved papillomavirus sequences from public domains including HPV Center (http://www.nordicehealth.se/hpvcenter/; accessed on 31 October 2018), Papillomavirus Episteme (PAVE, https://pave.niaid.nih.gov/; accessed on 31 October 2018), GenBank/NCBI (https://www.ncbi.nlm.nih.gov/; accessed on 31 October 2018), and our own PV database when this analysis was initiated (31 October 2018). We found that there were 782 complete genomes assigned with unique virus names as well as NCBI accession numbers. A pairwise sequence comparison using the complete L1 ORF nucleotide sequences identified 653 genomes sharing less than 90% similarity with other PV genomes and representing distinct types. Eleven HPV metagenomic sequences lacking complete ORFs of E1, E2, L1 or L2 gene, ScPV1 (accession number of MF564196) with unusual E6/E7 ORFs, and SaPV1 (KX643372) classified into the subfamily *Secondpapillomavirinae* (mainly containing fish PVs) were excluded from further analysis in this study, resulting in a total of 640 PV complete genomes (dataset 1, Table 1) (see PV list in Table S2). Among the analyzed PV genomes, 50 HPV types and 22 non-human primate PV types were characterized by the authors’ groups. In order to evaluate the specific characteristics and differences among HPV genera/groups, types, and genes, we refined a conserved dataset (dataset 2, N = 228) including 214 HPV complete genomes officially assigned by the HPV Center (65 Alpha-HPVs, 52 Beta-HPVs, 97 Gamma-HPVs) and 14 macaque PV types clustering into the genera Alpha-, Beta- and Gamma-PVs (Table S1). 

### 4.2. Alignment Based Phylogenetic Analysis 

Papillomavirus complete genome nucleotide sequences were linearized from the first ATG of the E1 ORF and aligned using MAFFT v7.221 [81]. The nucleotide sequences of each ORF were aligned using translation algorithm based on the aligned amino acid sequence matrix using MUSCLE v3.8.31 [82] within the Seaview v4.5.4 [83] program. Maximal likelihood (ML) trees were constructed using RAxML MPI v8.2.3 [84] with optimized parameters via CIPRES Science Gateway [85]. In order to detect phylogenetic incongruence among different genes, separated ML trees were constructed based on the concatenated nucleotide sequence alignments from the early genes (E6-E7-E1-E2 or E1-E2) or late genes (L2-L1). The tree topologies were illustrated using *plot.phylo* and *cophyloplot* function in R’s package ‘ape’ [86]. Papillomavirus classification and nomenclature were followed as previously described [7,8]. To compare genetic diversity between papillomavirus genera/groups, the *Pi* index [87] across the complete genomes or concatenated genes was calculated using DAMBE5 [88].

### 4.3. K-Mer Distribution Clustering 

The term *k*-mer refers to all subsequences of length *k* in a gene/genome, and for nucleotide sequence, there will be a total of 4*^k^* possible *k*-mers [39]. Given the relative short size of PV genome (~8000 bp), the trimer distributions (*k* = 3) composed of the frequencies of 64 3-bp forms across viral genomes were summarized to calculate a Kullback-Leibler (KL) distance matrix [89], based on which a hierarchical phylogeny was constructed. The *count* and *hclust* in R’s packages ‘seqinr’ [90] and ‘stats’ [91] were used to count the composition of trimers and construct the hierarchical tree, respectively.

### 4.4. Codon Usage Bias 

The effective number of codons (ENC) statistic is able to determine how biased a gene is in terms of its codon usage [48]. We used codonW package (http://codonw.sourceforge.net/; accessed on 31 October 2018) to calculate the ENC values that range between 20 (extremely strong bias as only a single codon for each amino acid is used) and 61 (no bias when a gene trends to use all codons with equal frequency). We further plotted the relationship between ENC and GC3s (GC content at the synonymous third codon position) to examine the influence of GC content on codon usage. This was compared to the expected ENC* if GC content were solely responsible for the codon biases, calculated as [42,48]:

$${\mathrm{ENC}}^{*}=2+\mathrm{GC}3s+\frac{29}{\mathrm{GC}3s^{2}+{\left(1-\mathrm{GC}3s\right)}^{2}}$$

### 4.5. Relative Synonymous Codon Usage (RSCU)

The ENC is widely used to characterize codon usage bias. However, it does not handle each codon differentially. We calculated the Relative Synonymous Codon Usage (RSCU) values for 59 codons (except for Met, Trp, and stop codons): the ratio of the observed frequency of codons relative to the expected frequency in the absence of usage bias to measure the extent of non-random usage of synonymous codons [92]. If the synonymous codons for an amino acid are equally used, the RSCU value would be 1. The value could be < 1 if an individual codon was less used than expected and vice versa. The website tool CAIcal (http://genomes.urv.cat/CAIcal/; accessed on 31 October 2018) was used to calculate the RSCU values. The RSCU values in the host genomes serve as the references, as retrieved from the Kazusa codon usage database (http://www.kazusa.or.jp/codon/; accessed on 31 October 2018) (*Homo sapiens*: 93487 CDs, 40662582 codons; *Macaca fascicularis*: 9092 CDs, 2451969 codons) [93]. 

### 4.6. Measuring of Dinucleotide Suppression

CpG suppression, the observation of lower-than-expected numbers of 5′-CG-3′ dinucleotides, is a common phenomenon in most portions of vertebrate genomes. This feature is likely to be due to C-to-T mutation, driven by CG-specific DNA methyl transferases. In viral genomes, suppression of CG dinucleotides may help them to evade host cellular defenses. We measured the dinucleotide suppression of the core dataset of 228 PV genomes including GpC and other 15 dimers, calculated as the observed frequency of the dinucleotide relative to the product of the frequencies of the individual nucleotides [94]. The dinucleotide O/E ratio would be 1 if the occurrences of its individual nucleotide were independent, and the genome exhibits suppression if it has XpY much less than 1. In double-stranded DNA (dsDNA) viruses, for dinucleotides that form a reverse complemented pair on the opposite strand (including ApT, CpG, GpC, TpA), we symmetrized the measure of CpG with the complementary dinucleotide as outlined by Burger et al. [95]:

$$\mathrm{CpG}=\frac{f_{\mathrm{CG}}}{f_{C}{\;*\;f}_{G}}$$

where f_CG_ represents the frequency of a dimer CG, f_c_ and f_G_ denote the probabilities of its constituent monomers, respectively. 

For dinucleotides that do not form a reverse complemented pair on the opposite strand (including ApA = TpT, ApC = GpT, ApG = CpT, CpA = TpG, CpC = GpG, GpA = TpC), for instance, the ratio is:

$$\mathrm{CpA}\left(=\mathrm{TpG}\right)=2*\frac{f_{\mathrm{CA}}+f_{\mathrm{TG}}}{\left(f_{C}+f_{G}\right)\;*\;\left(f_{A}+f_{T}\right)}$$

### 4.7. Correspondence Analysis

The correlation between codon usage and PV genomic composition was tested using correspondence clustering approaches including multidimensional scaling of the redundancy analysis (RDA) and optionally principal coordinate analysis (PCoA) [50]. The *rda* function in R’s package ‘vegan’ was used to generate two-dimensional representations for matrix 1 and 2 and visualized using the *biplot*. Differences in codon usage bias were assessed with permutational multivariate analysis of variance (PERMANOVA), using *adonis2* in R’s package ‘vegan’.

### 4.8. Statistical Analysis

The agreement between distance matrices inferred from sequence alignment and trimer distribution of PV genomes was measured using the Mantel test. The Pearson’s correlation coefficient was calculated to test the association between dinucleotide suppression pairs (*cor.test* in R’s package ‘stats’). The significance of the differences in the genetic metric measures between HPV genes and genomes was tested using phylogenetic generalized linear models (*pgls* function in R’s package ‘caper’). All plotting and statistical comparisons were performed using R scripts developed in house [91].

## Figures and Tables

**Table 1:** Relative synonymous codon usage (RSCU) pattern of the surveyed 228 human and macaque papillomaviruses (PVs) inferred from the concatenated 6 ORFs (E6-E7-E1-E2-L2-L1).

			Papillomaviruses							Host Species	
Amino Acid	Codon ^	All-HPV	All-PV	Alpha-HPV-HR	Alpha-HPV-LR1	Alpha-HPV-LR2	Beta-HPV	Gamma-HPV	Macaque-αPV	*Homo sapiens*	*Macaca fascicularis*
Ala (A)	GCA	1.70	1.62	2.00	1.92	1.66	1.65	1.64	1.64	0.91	1.04
	GCC	0.60	0.61	0.64	0.79	1.01	0.58	0.46	1.09	1.60	1.41
	GCG	0.28	0.31	0.25	0.34	0.42	0.27	0.24	0.44	0.42	0.36
	GCT	1.42	1.47	1.11	0.95	0.91	1.51	1.67	0.83	1.06	1.19
Cys (C)	TGC	0.62	0.69	0.50	0.73	0.96	0.69	0.51	1.04	1.09	0.99
	TGT	1.38	1.31	1.50	1.27	1.04	1.31	1.49	0.96	0.91	1.01
Asp (D)	GAC	0.68	0.71	0.76	0.88	0.98	0.68	0.56	1.05	1.07	0.98
	GAT	1.32	1.29	1.24	1.12	1.02	1.32	1.44	0.95	0.93	1.02
Glu (E)	GAA	1.33	1.25	1.36	1.26	1.05	1.31	1.41	0.88	0.84	0.95
	GAG	0.67	0.75	0.64	0.74	0.95	0.69	0.59	1.12	1.16	1.05
Phe (F)	TTC	0.25	0.33	0.11	0.14	0.36	0.25	0.26	0.44	1.07	0.96
	TTT	1.75	1.67	1.89	1.86	1.64	1.75	1.74	1.56	0.93	1.04
Gly (G)	GGA	1.27	1.28	1.05	1.09	0.90	1.31	1.43	0.74	1.00	1.15
	GGC	0.73	0.71	0.86	0.88	1.04	0.76	0.58	1.26	1.35	1.20
	GGG	0.73	0.79	0.74	0.79	1.10	0.73	0.61	1.25	1.00	0.93
	GGT	1.27	1.22	1.36	1.24	0.96	1.20	1.38	0.75	0.65	0.73
His (H)	CAC	0.56	0.62	0.62	0.69	0.88	0.48	0.47	1.11	1.16	1.06
	CAT	1.44	1.38	1.38	1.31	1.12	1.52	1.53	0.89	0.84	0.94
Ile (I)	ATA	1.34	1.29	1.52	1.45	1.49	1.24	1.29	1.51	0.51	0.58
	ATC	0.22	0.31	0.10	0.15	0.28	0.29	0.22	0.32	1.41	1.24
	ATT	1.43	1.40	1.38	1.40	1.23	1.47	1.49	1.18	1.08	1.18
Lys (K)	AAA	1.43	1.38	1.51	1.31	1.11	1.42	1.53	0.99	0.87	0.94
	AAG	0.57	0.62	0.49	0.69	0.89	0.58	0.47	1.01	1.13	1.06
Leu (L)	CTA	0.74	0.72	0.86	0.89	1.02	0.65	0.65	0.75	0.43	0.48
	CTC	0.17	0.26	0.07	0.06	0.21	0.25	0.15	0.30	1.17	1.06
	CTG	0.65	0.80	0.51	0.69	1.27	0.62	0.53	1.78	2.37	2.08
	CTT	0.80	0.89	0.56	0.57	0.70	0.87	0.88	0.58	0.79	0.92
	TTA	2.40	2.11	2.80	2.60	1.43	2.36	2.57	1.16	0.46	0.57
	TTG	1.24	1.22	1.20	1.20	1.37	1.25	1.22	1.43	0.77	0.88
Asn (N)	AAC	0.54	0.60	0.59	0.66	0.77	0.51	0.47	1.06	1.06	0.98
	AAT	1.46	1.40	1.41	1.34	1.23	1.49	1.53	0.94	0.94	1.02
Pro (P)	CCA	1.40	1.35	1.43	1.18	1.07	1.47	1.48	0.91	1.11	1.22
	CCC	0.56	0.62	0.55	0.61	0.96	0.55	0.45	1.41	1.29	1.15
	CCG	0.25	0.30	0.22	0.30	0.36	0.24	0.23	0.37	0.45	0.39
	CCT	1.79	1.73	1.80	1.91	1.60	1.74	1.85	1.31	1.15	1.25
Gln (Q)	CAA	1.19	1.13	1.19	1.14	0.82	1.29	1.25	0.80	0.53	0.61
	CAG	0.81	0.87	0.81	0.86	1.18	0.71	0.75	1.20	1.47	1.39
Arg (R)	AGA	2.35	2.39	1.87	1.67	1.22	2.28	2.92	1.33	1.29	1.53
	AGG	1.01	1.07	1.06	1.21	1.21	1.21	0.80	1.28	1.27	1.29
	CGA	0.82	0.78	0.75	0.66	0.66	0.85	0.88	0.55	0.65	0.69
	CGC	0.58	0.59	0.57	0.78	0.99	0.55	0.45	1.02	1.10	0.89
	CGG	0.42	0.44	0.47	0.59	0.77	0.46	0.27	0.81	1.21	1.07
	CGT	0.83	0.72	1.27	1.09	1.15	0.66	0.68	1.01	0.48	0.52
Ser (S)	AGC	0.75	0.88	0.67	0.67	1.09	0.75	0.69	1.71	1.44	1.28
	AGT	1.56	1.45	1.91	1.71	1.46	1.39	1.58	1.04	0.90	1.00
	TCA	1.13	1.12	1.08	1.07	0.75	1.25	1.20	0.87	0.90	0.99
	TCC	0.77	0.75	0.65	0.83	1.15	0.91	0.62	1.08	1.31	1.20
	TCG	0.25	0.28	0.20	0.21	0.32	0.26	0.24	0.31	0.33	0.29
	TCT	1.53	1.52	1.49	1.51	1.24	1.44	1.68	1.00	1.13	1.24
Thr (T)	ACA	1.70	1.63	2.00	2.00	1.59	1.68	1.63	1.42	1.14	1.24
	ACC	0.70	0.72	0.65	0.69	1.06	0.79	0.58	1.10	1.42	1.28
	ACG	0.26	0.29	0.28	0.36	0.42	0.21	0.23	0.59	0.46	0.40
	ACT	1.33	1.37	1.08	0.95	0.93	1.31	1.57	0.89	0.99	1.08
Val (V)	GTA	1.34	1.22	1.60	1.42	1.15	1.30	1.35	0.82	0.47	0.56
	GTC	0.31	0.39	0.16	0.18	0.32	0.41	0.30	0.27	0.95	0.88
	GTG	1.07	1.10	1.15	1.36	1.75	0.99	0.86	2.15	1.85	1.71
	GTT	1.28	1.29	1.09	1.03	0.78	1.31	1.49	0.77	0.73	0.85
Tyr (Y)	TAC	0.43	0.52	0.33	0.36	0.52	0.44	0.42	0.69	1.11	1.02
	TAT	1.57	1.48	1.67	1.64	1.48	1.56	1.58	1.31	0.89	0.98

^ The preferred codons (RSCU > 1.6) and the suppressed codons (RSCU < 0.6) are highlighted in green and red, respectively.

**Table 2:** Dinucleatide suppression in the surveyed human and macaque papillomavirus genomes inferred from the concatenated 6 ORFs.

Amino Acid	All-HPV	All-PV	Alpha-HPV-HR	Alpha-HPV-LR1	Alpha-HPV-LR2	Beta-HPV	Gamma-HPV	Macaque-αPV
ApA (=TpT)	1.04	1.04	0.99	1.00	1.00	1.05	1.06	1.04
ApC (=GpT)	0.98	0.98	1.11	1.06	1.04	0.93	0.94	1.01
ApG (=CpT)	1.03	1.03	0.92	0.94	0.97	1.07	1.07	0.99
ApT	0.96	0.96	1.00	1.00	1.00	0.95	0.93	0.96
CpA (=TpG)	1.23	1.24	1.30	1.31	1.30	1.21	1.20	1.36
CpC (=GpG)	1.14	1.14	1.20	1.19	1.18	1.13	1.11	1.13
CpG	0.48	0.48	0.47	0.48	0.54	0.48	0.46	0.52
GpA (=TpC)	0.88	0.87	0.72	0.72	0.75	0.94	0.95	0.74
GpC	1.08	1.08	1.06	1.11	1.06	1.06	1.09	1.12
TpA	0.89	0.89	1.00	0.98	0.96	0.85	0.85	0.86

## Data Availability

All sequences analysed in this study are available in the NCBI GenBank database, with accession numbers listed in Table S2.

## Supplementary Materials

The following are available online at https://www.mdpi.com/article/10.3390/ijms22179657/s1.
