# Supplementary material for: K-Mer Analyses Reveal Different Evolutionary Histories of Alpha, Beta, and Gamma Papillomaviruses
Source: Int J Mol Sci. 2021 Sep 6;22(17):9657. doi: 10.3390/ijms22179657 (PMC8432194; doi:10.3390/ijms22179657)
Supplement: Supplementary file 1 [file ijms-22-09657-s001.zip › ijms-1362808-supplementary.pdf]

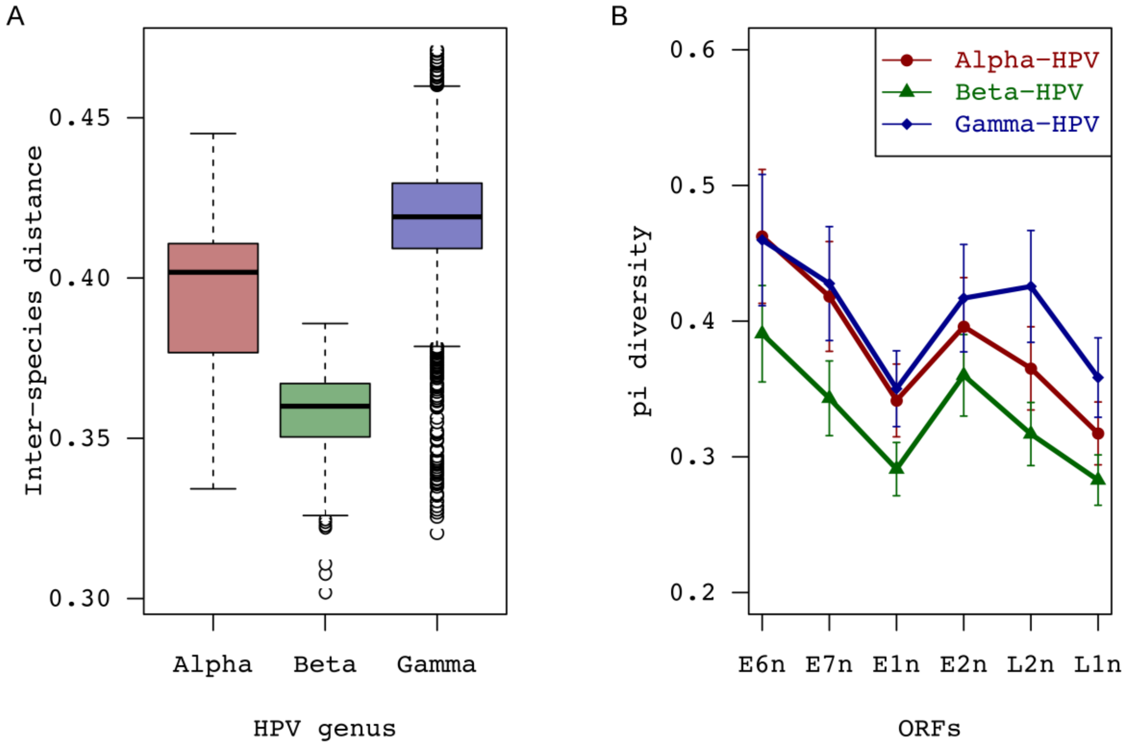

Figure S1. Genetic diversity of Alpha-, Beta- and Gamma-PV types. (A) Boxplot of distances between HPV species of the genera Alpha-, Beta- and Gamma-PV genus. (B) The pi diversities of HPV genes within the genera Alpha-, Beta- and Gamma-PV.

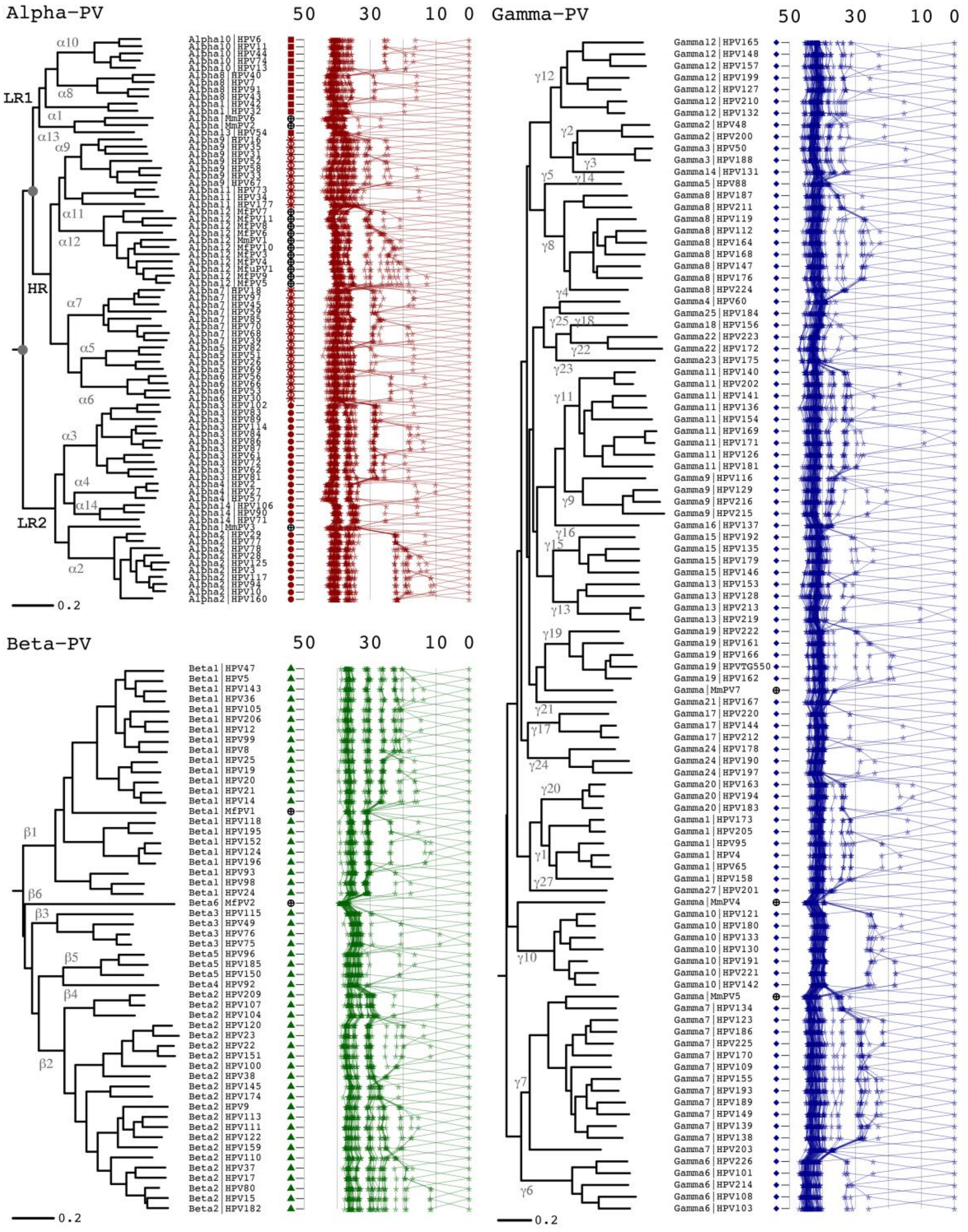

Figure S2. Tree topologies and pairwise comparisons of Alpha-, Beta- and Gamma-PV types. The maximum likelihood (ML) tree was constructed using RAxML MPI v7.2.8 inferred from the concatenated nucleotide sequence alignments of E6-E7-E1-E2-L2-L1 ORFs of PV types within the genera Alpha-, Beta- and Gamma-PV. The bar at the bottom of the trees indicates the nucleotide substitution of unit changes (i.e., 0.2) per site. The percent nucleotide differences for PV types within each genus are shown in the panel to the right of the phylogeny. These represent each genome compared to all other genomes, with comparison to self being 0% different.

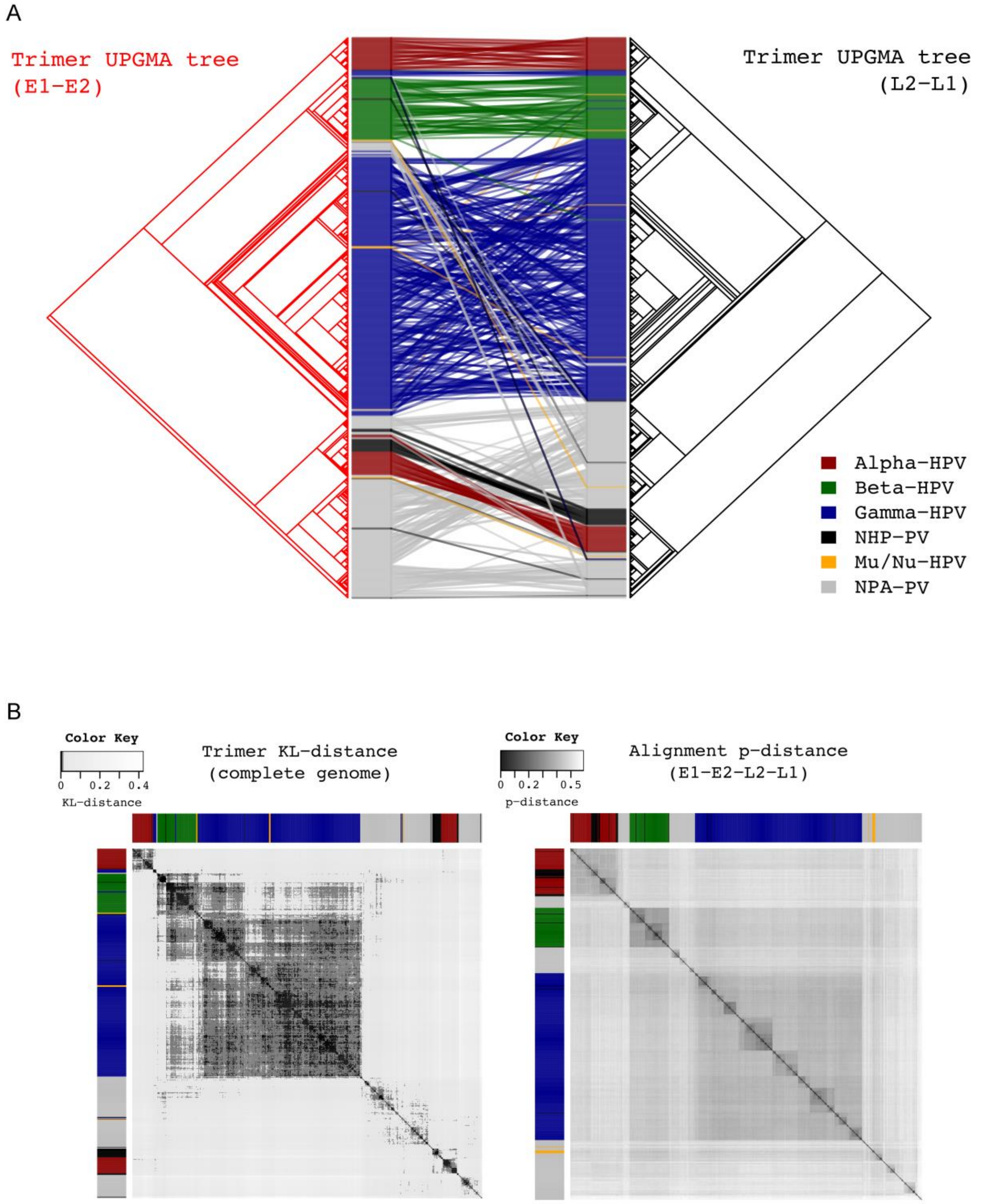

Figure S3. Tanglegram of early and late genes and heat maps of trimer and homology analyses. (A) Tanglegram of tree topologies between the hierarchical clustering of trimer distributions based on the concatenated nucleotide sequence alignments of E1-E2 and L2-L1 ORFs of 640 PV types. (B) Heat map of PV trimer distributions. The proportional abundances (%) of each trimer (n=64 possible trimers) in 640 PV types were constructed based on the complete genome nucleotide sequences as a heat map scaled in color gradients, with white indicating minimal probability (0.14%) and black indicating the maximum probability (4.83%). The dendrogram was generated using hierarchical clustering based on trimer distributions KL distances. (C) Heat map of PV p-values of aligned sequences based on RAxML topology.

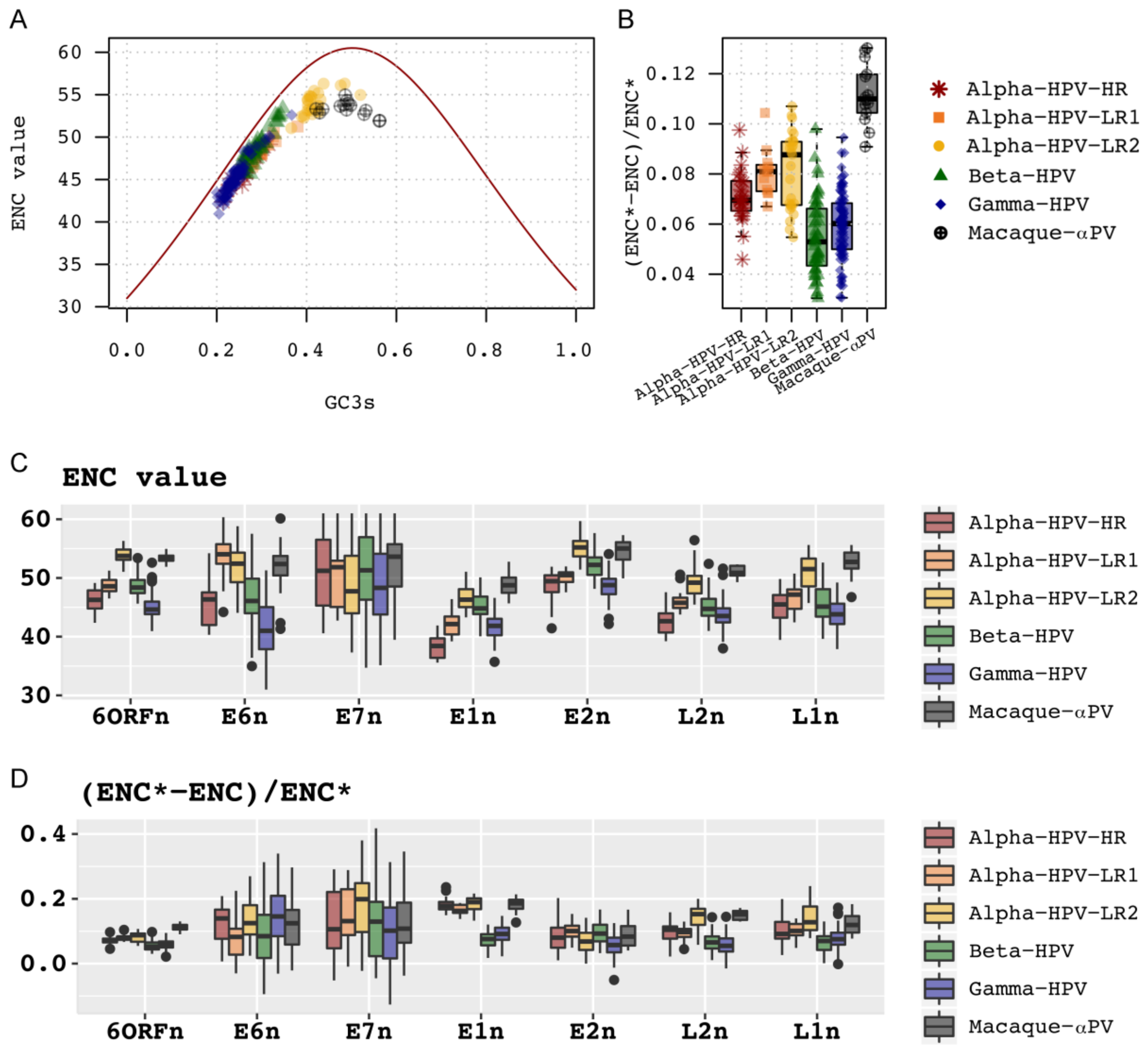

Figure S4. Synonymous codon usage of 228 PV genomes. (A) Plot of ENC and the synonymous third codon position (GC3s) content based on the concatenated nucleotide sequences of 6 ORFs (E6-E7-E1-E2-L2-L1). The red curve indicates the expected ENC* if codon usage pattern is only affected by GC3s. (B) Boxplot of the difference between the observed and expected ENC values between PV groups/genera. (C) Boxplot of Effective Number of Codon (ENC) and the difference between observed ENC and expected ENC* inferred from PV genes within each PV group/genus. (D) Boxplot of the difference between the observed and expected ENC values between PV groups/genera by ORF.

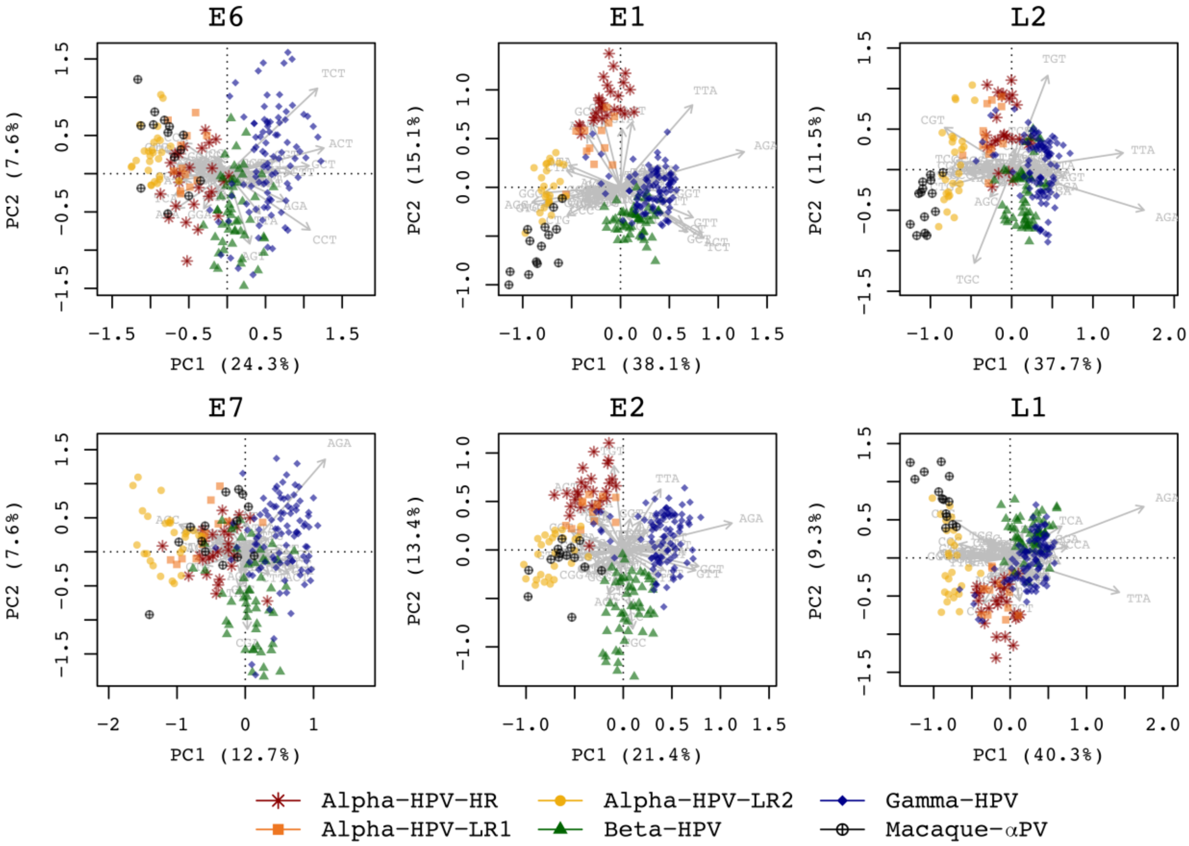

Figure S5. Scatter biplot of RSCU difference of 228 PV types inferred from each PV gene. The clustering was performed using redundancy analysis (RDA), with colors assigned to different PV groups/genera. The x-axis and the y-axis represent the first two principal coordinate component (PC) axes.

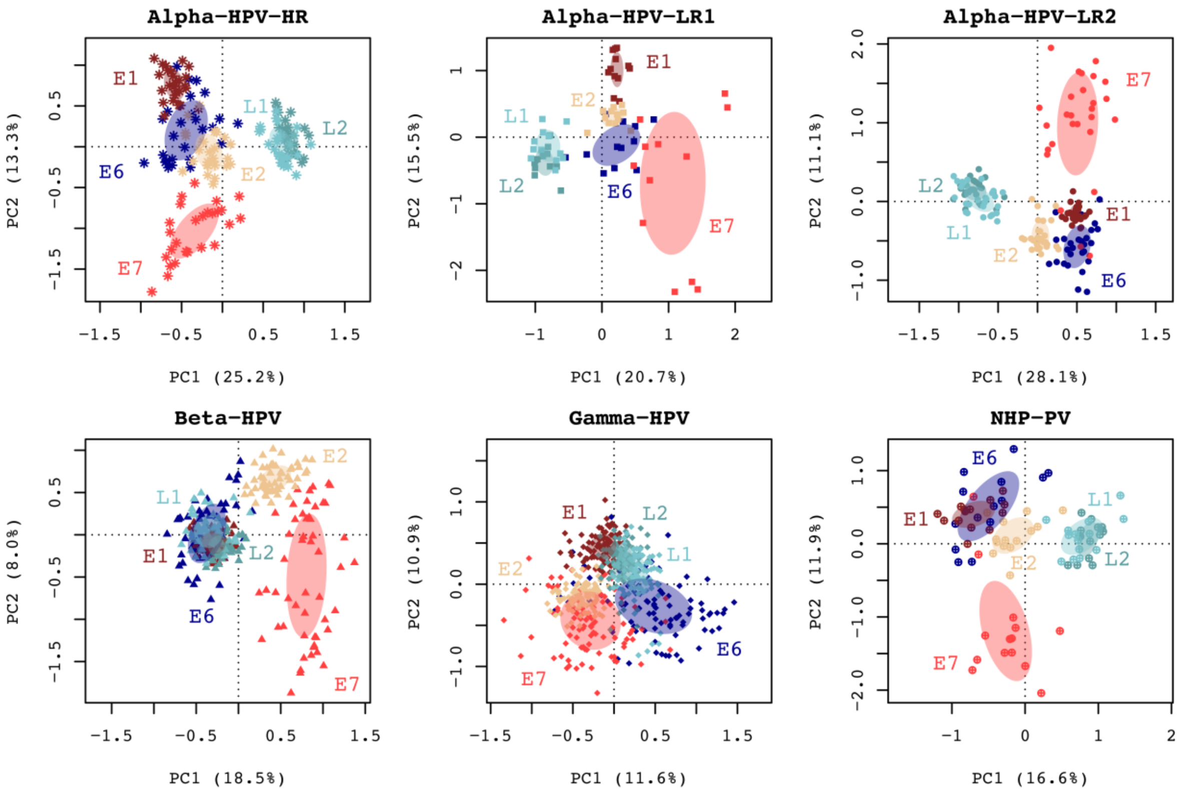

Figure S6. Scatter plot of RSCU difference of PV genes within each PV group/genus. Redundancy analysis was performed based on RSCU patterns for individual gene of 228 PV type; colors were assigned to each ORF, as represented by each symbol. The x-axis and the y-axis represent the first two principal coordinate component (PC) axes.

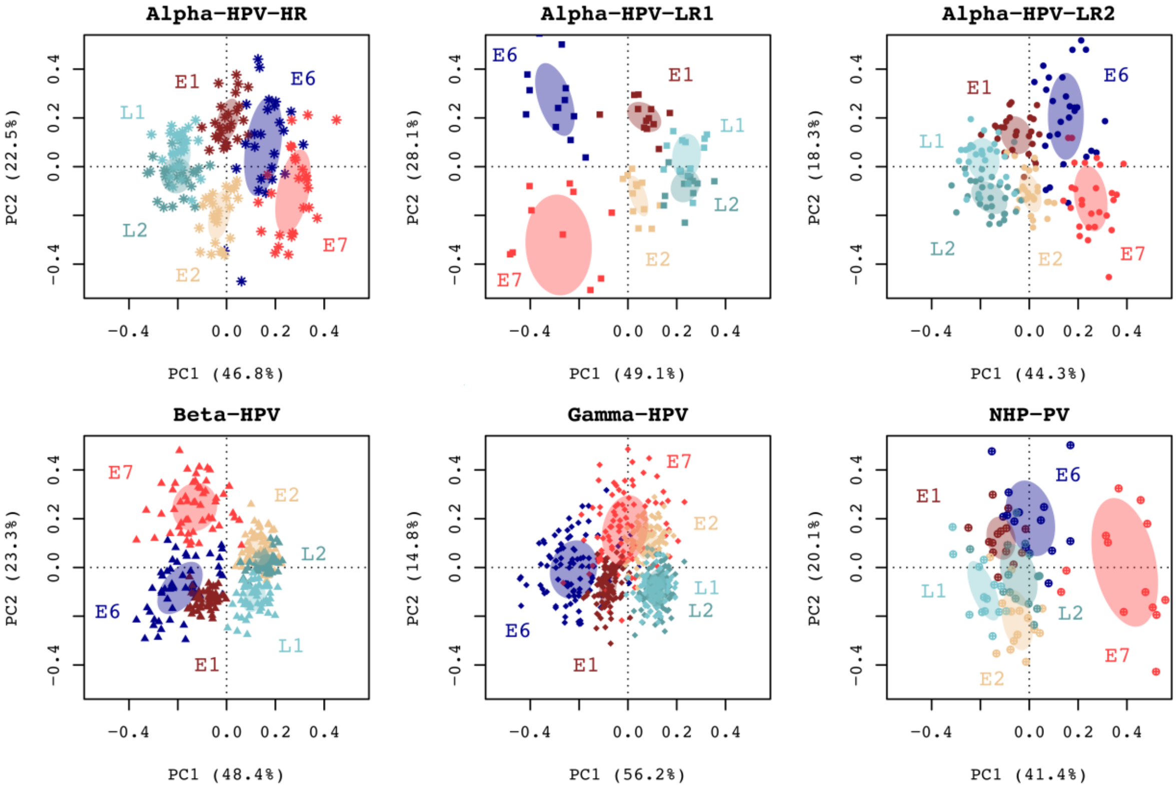

Figure S7. Scatter plot of relative abundance of dinucleotides of PV genes within each PV group/genus. Redundancy analysis was performed based on the patterns of relative abundance of dinucleotides for individual gene of 228 PV type; colors were assigned to each ORF, as represented by each symbol. The x-axis and the y-axis represent the first two principal coordinate component (PC) axes.

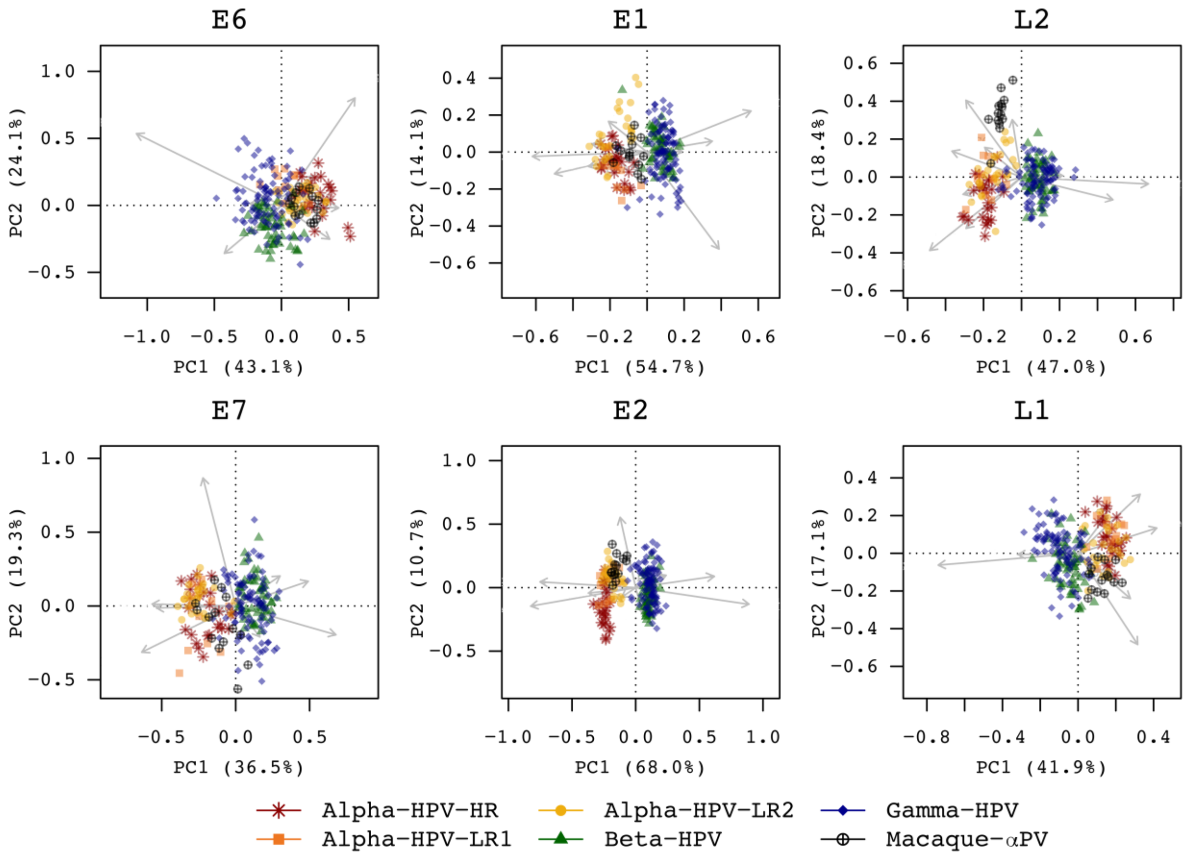

Figure S8. Scatter plot of relative abundance of dinucleotides of 228 PV types inferred from each PV gene. The clustering was performed using redundancy analysis (RDA), with colors assigned to different PV groups/genera. The x-axis and the y-axis represent the first two principal coordinate component (PC) axes.
